# Draft Genome Sequence of the Freshwater Diatom Fragilaria crotonensis SAG 28.96

**DOI:** 10.1128/mra.00289-22

**Published:** 2022-08-17

**Authors:** Brittany N. Zepernick, Alexander R. Truchon, Eric R. Gann, Steven W. Wilhelm

**Affiliations:** a Department of Microbiology, University of Tennessee, Knoxville, Tennessee, USA; University of California, Riverside

## Abstract

Here, we report the assembled and annotated genome of the freshwater diatom Fragilaria crotonensis SAG 28.96. The 61.85-Mb nuclear genome was assembled into 879 contigs, has a GC content of 47.40%, contains 26,015 predicted genes, and shows completeness of 81%.

## ANNOUNCEMENT

Fragilaria crotonensis is broadly distributed in freshwater systems, including both oligotrophic and hypereutrophic lakes, and serves as a biological indicator of eutrophication (1–5). F. crotonensis is an important member of Lake Erie’s phytoplankton because it has historically bloomed in summer (6) and remains a dominant member seasonally (7–11). To facilitate diatom-focused omics studies of Lake Erie and other lakes, we report the assembled and annotated F. crotonensis SAG 28.96 genome. The 61.85-Mb genome was assembled into 879 contigs, with 26,015 predicted genes and a GC content of 47.40%. The genome is predicted to be 81% complete (Table 1).

**TABLE 1:** General features of the F. crotonensis SAG 28.96 nuclear genome

Parameter^*a*^	Finding for Fragilaria crotonensis
Genome size (Mb)	61.85
GC content (%)	47.40
No. of contigs	879
*N*_50_ (bp)	89,148
*L*_50_ (contigs)	206
Total no. of predicted genes	26,015
No. of annotated genes	11,422
No. of unannotated genes	14,593
Avg gene length (bp)	1,283.73
Coding density	0.54
Completeness (%)	81
Sequencing depth (×)	58

a Genome size, GC content, number of contigs, and *N*_50_ and *L*_50_ values were determined via tQUAST-LG (v5.0.2). Genome completeness was assessed via BUSCO (v5.2.2) using the Stramenopile markers data set. Coding density is defined as follows: ([average gene length [bp] × total number of genes]/genome size [bp]). Sequencing depth is defined as follows: (total number of pooled reads [bp]/genome size [bp]).

Nonaxenic unialgal cultures of F. crotonensis SAG 28.96 (Culture Collection of Algae at the University of Göttingen, Göttingen, Germany) were cultured and collected as reported previously (8). DNA was extracted using standard phenol-chloroform methods with ethanol precipitation (12) and was quantified using the Qubit double-stranded DNA (dsDNA) HS assay kit (Invitrogen). Short-read sequencing was performed using an Illumina NovaSeq 6000 system (65 million paired-end 250-bp reads) at the Clinical Genomics Center (Oklahoma Medical Research Foundation, Oklahoma City, OK) with libraries prepared using the Illumina TruSeq PCR-free LT kit (350-bp insert). Long-read sequencing was performed in-house using a MinION MK1B R9.4.1 flow cell (*N*_50_, 17.815 kb; total number of reads, 642,517; total read length, 5.38 Gb) with high-molecular-weight DNA prepared with the ligation sequencing kit SQK-LSK109 (Oxford Nanopore Technologies) (13).

Assembly and gene prediction were performed using a previously established pipeline (14). Briefly, bases were called for Nanopore reads with Guppy (v4.0.15) (15). Adapters were trimmed using Porechop (v0.2.4) (16) with reads trimmed for quality (Q scores of 9) and length (500 bp) using NanoFilt (v2.7.1) (17). Illumina reads were trimmed using CLC Genomics Workbench (v20.0, with default settings). The assembly was performed using Canu (v2.1) (18). Contigs were polished using Pilon (v1.23) (19) with read mappings generated using Bowtie2 (v2.2.3) (20). Redundant contigs due to heterogeneity in diploid genomes were removed using Redundans (v0.14a) (21). Removal of bacterial contamination was performed using the Kaiju web server (22). Genome completeness was assessed by BUSCO (v5.2.2) using the Stramenopile database (23). Genes were called using BRAKER (24) with F. crotonensis transcriptomic data (25) that were assembled in CLC Genomics Workbench and mapped to the assembly using Hisat2 (26). Translated amino acid sequences were uploaded to the eggNOG-mapper web server to predict function (27). Contigs lacking coding sequences or those containing only bacterial genes were removed, along with the organellular genomes. tRNAs were predicted using tRNA-scan-SE (v2.0.6) (28). Genome statistics were determined using QUAST-LG (v5.0.2) (29).

**FIG 1:**
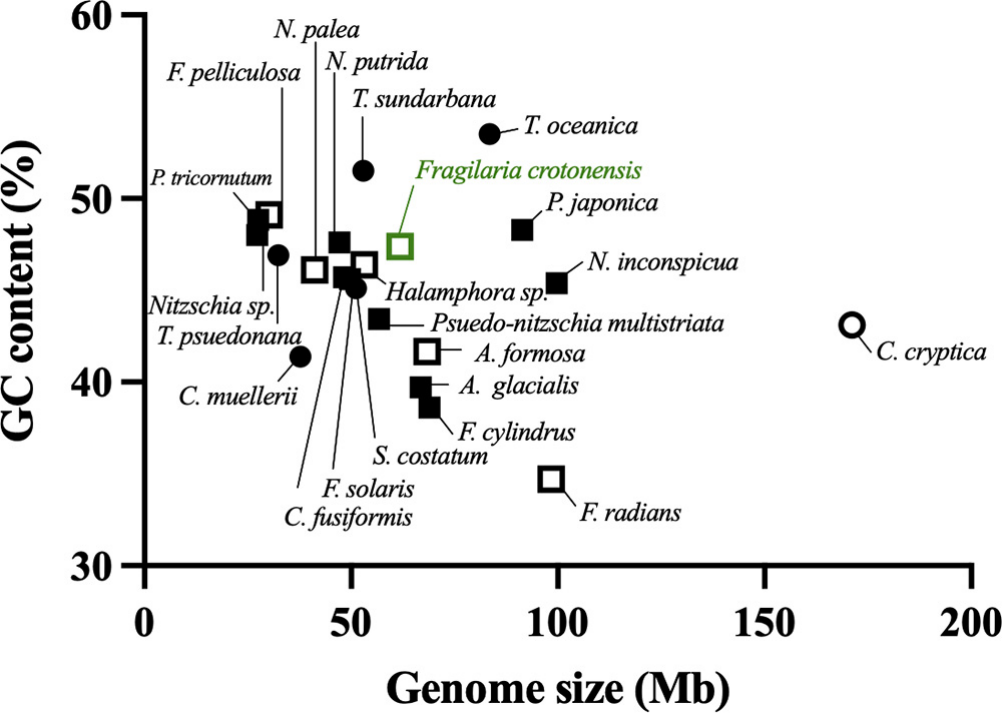
Variability of genome size and GC content of 21 Bacillariophyta genomes sequenced, annotated, and available to date in the NCBI taxonomy database, in addition to the newly sequenced F. crotonensis genome. Diatoms classified as estuarine/marine are indicated by filled symbols (*n* = 15), while freshwater diatoms are indicated by open symbols (*n* = 7). Centric diatoms are indicated by circles (*n* = 6), while pennate diatoms are indicated by squares (*n* = 16). The genome of F. crotonensis SAG 28.96 is indicated in green. An unclassified Bacillariophyta genome and a Licmophora abbreviata (environmentally assembled sample) genome are not included in this graph.

Until recently, diatom research primarily relied on two model marine diatom genomes (30, 31). There are now 22 fully characterized Bacillariophyta genomes available, but only 6 are freshwater (Fig. 1). A lack of representative freshwater diatom genomes is a gap in the field because differences in physiology exist. There are further morphological distinctions stemming from evolutionary divergence. As a result, there is a need to sequence not only freshwater diatom taxa but also a greater variety of morphologically and evolutionarily distinct diatoms to facilitate future diatom omics studies.

### Data availability.

The annotated nuclear genome was deposited in GenBank under the accession number JAKSYS000000000. Data are available under BioProject accession number PRJNA807324 and BioSample accession number SAMN25978007.
